# Improving adherence towards bowel preparation for colonoscopy

**DOI:** 10.1002/jgh3.12975

**Published:** 2023-09-23

**Authors:** Sanjiv Mahadeva

**Affiliations:** ^1^ Division of Gastroenterology, Department of Medicine University of Malaya Kuala Lumpur Malaysia

Colorectal cancer (CRC) is a significant health problem globally. The Asia‐Pacific region has the largest number of cases of CRC and one of the highest levels of mortality due to this condition in the world.^1^ However, if diagnosed at an early stage, CRC is one of the most preventable and curable malignancies. Current recommendations on colorectal screening advocate colonoscopy as the preferred modality because of its accuracy in detecting early lesions and proven efficacy in lowering rates of incident CRC.^1^ Lesions characterized as early cancers can be endoscopically resected at the same sitting and can be curative.^2^ Despite these advances in diagnostic and therapeutic colonoscopy, the utility of colonoscopy remains dependent on the cleanliness of the colon or the quality of bowel preparation.

Poor bowel preparation has been shown to lead to a lower adenoma detection rate, reduced cecal intubation rate, prolonged cecal intubation and total colonoscopy time, and increased patient discomfort.^3^, ^4^ A good bowel preparation is therefore essential for colonoscopy. Several colon cleansing agents and schedules have been used and studied for bowel preparation during colonoscopy. The most popular regimes today are based on polyethylene glycol (PEG)–electrolyte lavage solution.^5^ PEG is a non‐absorbable solution that should pass through the bowel without net absorption or secretion. Significant fluid and electrolyte shifts are therefore avoided, but large volumes (4 L) are still required to achieve a cathartic effect. These large volumes of fluid consumption in a single setting commonly cause nausea and vomiting in patients, often leading to poor adherence and noncompletion of the PEG preparation. To improve the adherence to PEG solutions, reduced volume (2 L) preparations coupled with irritant laxatives such as bisacodyl or magnesium citrate have been developed to increase patient compliance and are recognized to be as effective as the standard 4 L PEG preparation.^6^ An alternative approach has been to split the 4 L PEG dosing to the day before and on the day of the procedure, which has been suggested to reduce adverse gastrointestinal (GI) symptoms and improve adherence.^7^

In this issue of *JGH OPEN*, Yang et al. have reported a randomized trial comparing predominantly 4 L PEG solution and 2 L PEG in combination with Linaclotide as bowel preparation for colonoscopy in 266 patients.^8^ There was no difference in bowel preparation quality or colon polyp detection rate between the two groups. However, there was better sleeping quality and a lower rate of GI symptoms (nausea, vomiting, abdominal bloating) in the 2 L PEG + Linaclotide compared with the 4 L PEG group. Linaclotide, a guanylate cyclase‐C (GC‐C) agonist, which increases intestinal chloride and fluid secretion by activating the guanosine cyclic phosphate (cGMP) cascade, has been shown to be effective in the treatment of constipation‐predominant irritable bowel syndrome (IBS‐C) and chronic idiopathic constipation (CIC).^9^

This study provides yet another option for a more palatable bowel preparation, which will help increase adherence by patients, leading to a better quality colonoscopy examination. However, Linaclotide is still not widely available in the Asian region as a therapy for even IBS/constipation,^10^ let alone for bowel preparation. For the time being, enhancing adherence can include both combining low‐volume 2 L PEG + laxatives together with splitting the dose. A recent study that compared same‐day *versus* split‐dosing of 2 L PEG + bisacodyl found no difference in bowel preparation quality, but lower GI symptoms and greater adherence with the latter.^11^

Lastly, it is important to remember that the type and timing of bowel preparation are not the only factors influencing the quality of bowel preparation. Extrinsic factors such as patient education, appointment reminders, and reducing the waiting times of colonoscopy appointments have been shown to improve adherence to bowel preparation and ultimately to a better quality colonoscopy.^12^, ^13^
